# Footcare Intervention Delivered by Community Health Workers: A Modified Delphi Study

**DOI:** 10.7759/cureus.70580

**Published:** 2024-09-30

**Authors:** Okatiranti Okatiranti, Richard Windle, Henry B Perry, Sarah Goldberg

**Affiliations:** 1 Department of Nursing, Adhirajasa Reswara Sanjaya (ARS) University, Bandung, IDN; 2 School of Health Sciences, University of Nottingham, Nottingham, GBR; 3 School of Public Health, John Hopkins University, Baltimore, USA

**Keywords:** community health workers, core components of intervention, delphi study, diabetes foot ulcers (dfus), footcare

## Abstract

Studies have shown that community health workers (CHWs) can effectively deliver footcare interventions (FCIs) as part of diabetes self-management education programmes in high-resource countries. However, more evidence is needed on implementing FCI in low-resource countries and more detailed information about the core components of FCI provided by CHWs. This study aims to refine the core components of the footcare intervention to be delivered by CHWs (FIne-CHWs) for T2DM patients and to analyse Delphi panel members’ opinions and perceptions regarding the core components of FIne-CHWs. Triangulation data from previous steps of data collection (scoping and mapping review combined with interview data) identified 42 statements representing the core components of FIne-CHWs. After two rounds of a Delphi exercise involving 22 panel members, experts in diabetes from all over Indonesia, a consensus agreement was reached on 41 out of 42 statements of the core components of FIne-CHWs. Free-text responses to the Delphi survey were analysed using thematic analysis. One overarching theme emerged: CHWs “are only volunteers.” This then was discussed in three themes, namely CHWs deliver footcare education for all people with diabetes, remuneration and incentives, and flexibility of community FCI. This study determined the core components of FIne-CHWs and also identified that the position of CHWs within the Indonesian healthcare system could impact the implementation of the FIne-CHWs programme.

## Introduction

Diabetic foot ulcers (DFUs) are commonly observed in diabetic patients worldwide [1], including in Indonesia [2], and must never be neglected due to their high associated mortality rate [3]. About 33.9% of all diabetic patients hospitalised in 2007 were due to DFUs and associated gangrene in one National General Hospital. Fourteen per cent of these patients died, and 35% had major complications or minor amputations [2].

One potential approach to reducing DFU incidence is empowering the community to increase awareness about diabetes prevention and promotion. Community health workers (CHWs) delivering footcare interventions (FCIs) have shown promise in terms of improving diabetes foot management and related health outcomes in medically underserved communities [4]. CHWs, known locally as kader, handle primary healthcare tasks like maternal health, immunisations, and tuberculosis medication adherence. In Indonesia, community posts or integrated service posts (ISP) are managed by CHWs, such as ISPs for children and maternal (Posyandu) and for managing non-communicable diseases (NCDs) (Posbindu PTM). The selection criteria for becoming a CHW include the ability to read and write, possessing a sociable and voluntary spirit, having knowledge of the community's customs and habits, a willingness to commit the necessary time, residing in the village, being friendly and empathetic, and being accepted by the community. Although being female is not a requirement, the majority of volunteers are women [5]. CHWs who manage ISP-NCDs are required to have 12 years of education [6]. However, the new guidance for CHWs reported that no educational attainment is required to become a CHW [7]. CHWs are expected to provide health promotion for diabetes patients to prevent the occurrence of DFUs. They work voluntarily, supervised by healthcare professionals, but are primarily accountable to the village committee under the Ministry of Home Affairs (MoHA) [5].

This study aimed to refine the core components of a footcare intervention to be delivered by CHWs (FIne-CHWs) for T2DM patients in Indonesia and to analyse Delphi panel members’ opinions and perceptions regarding the core components of FIne-CHWs.

This article was previously published as a thesis for the degree of Doctor of Philosophy at the University of Nottingham on 19th July 2024 [8] and was presented at the AH PGR Conference at the Monica Partridge Building, University of Nottingham, on 24th January 2023.

## Materials and methods

A three-round modified Delphi approach was used to gain consensus on the core components of FIne-CHWs. The Delphi technique is a method pertaining to the utilisation of expert opinions [9]. The Delphi statements were developed using multiple methodological approaches, including scoping and mapping reviews and key stakeholder interview data. The data from the previous steps were separately analysed before the process of triangulation was employed [10].

The 42 statements were generated from a triangulation protocol and posed to the Delphi panel in the first round of the process to gain panel consensus agreement or disagreement (Table 1). The statements were grouped based on a template for intervention description and replication (TIDieR) guidelines [11], which encompass the core components of the intervention identified, including items on intervention procedures (n=3), intervention providers (n=16), place of intervention (n=2), delivery methods (n=7), timing and duration (n=3), educational content (n=9), and tailored intervention (n = 2) (Table 1).

**Table 1:** First round of Delphi consensus questionnaire FIne-CHWs: footcare intervention to be delivered by community health workers, CHWs: community health workers, HCPs: healthcare professionals, Posbindu PTM: integrated services post for non-communicable disease, DFUs: diabetic foot ulcers

Item no.	Core components of FIne-CHWs
Intervention procedures	
1	Only patients without an active DFU are eligible for the FIne-CHWs sessions
2	A registered healthcare professional (such as a nurse, physician or podiatrist) must screen all patients’ feet for active DFUs before referring them to the FIne-CHWs educational sessions
3	All patients should have their blood glucose levels measured within one month before attending the first FIne-CHWs session
Intervention providers (CHWs)	
4	CHWs delivering the FIne-CHW sessions must have passed senior high school
5	CHWs with the right personal specifications can be trained to deliver the FIne-CHWs educational sessions competently
6	CHWs delivering the sessions must not have any political affiliations
7	CHWs delivering the FIne-CHW sessions must have good communication skills and be motivated to deliver diabetic footcare Intervention
8	CHWs must be trained on DFU care before they can deliver the FIne-CHWs sessions
9	The curriculum for training CHWs should also cover how to treat patients with dignity, compassion, and respect
10	CHWs should be provided a course manual detailing how they should deliver the sessions
11	The training of CHWs should use the teach-back method to emphasise practical skills
12	The CHWs' knowledge, skills, and attitudes must be tested through an examination prior to them providing training to patients
13	The CHWs providing the FIne-CHW sessions must be under the management of the community health centre or Posbindu PTM
14	There must be a policy for referring patients to the FIne-CHWs
15	CHWs must be reimbursed for their travel costs to the community centre, Posbindu PTM, or patients’ homes
Intervention providers (HCPs)	
16	CHWs providing the FIne-CHWs sessions should be supervised by general practitioners or registered nurses
17	A registered nurse should be present at the community centre or Posbindu PTM to supervise CHWs whilst they are delivering the sessions
18	The nurse supervising the CHWs delivering the session should have previously received training in the care of patients with DFUs
19	Footcare training for nurses should also emphasize foot examination to categorize the risk of DFUs
Intervention place	
20	The FIne-CHW intervention should be delivered in the community centre such as the Posbindu PTM or delivered at the community health centre unless the patient is assessed as needing a home visit
21	Where patients are assessed as unable to visit the Posbindu PTM (e.g., due to physical mobility issues), the CHWs should provide the intervention in the patient’s own home, with booster sessions provided by telephone
Method of delivery	
22	FIne-CHW sessions should be in groups of up to 10 patients and family members
23	Family members should be actively encouraged to attend the FIne-CHW sessions with the patient
24	FIne-CHW sessions should always be delivered by two CHWs: one delivers educational materials whilst the other monitors patients’ understanding and provides further explanations
25	The educational sessions should consist of multiple teaching and learning methods such as lectures, group discussions, video presentations, and hands-on skill sessions
26	The FIne-CHWs sessions should include a video on diabetic footcare from a diabetes expert
27	All patients attending the FIne-CHW sessions should be provided with educational printed materials (such as illustrated pamphlets) to take home
28	The CHWs should be provided with the foot self-care kit containing a basin (patients should bring their own basin), a gallon of water, antibacterial, nondeodorant soap, a hand towel, a washcloth, an emery board, hypoallergenic lotion, and a mirror
Time and duration	
29	FIne-CHW sessions should be provided in four sessions: two one-hour educational sessions and two 15-30-minute booster sessions
30	The booster sessions will reinforce the teaching and learning from the first two sessions and the need for patients to perform daily footcare
31	The FIne-CHW sessions should be given over a six-month period
Intervention content	
32	Educational content should cover knowledge on awareness about diabetes and foot complications: definition of the diabetic foot, its types, warning signs, footcare, etc.
33	Washing feet
34	Inspecting foot for problems
35	Moisturizing and massaging foot
36	Foot exercise (e.g., foot exercise using newspaper/papers)
37	Toenail care
38	Wearing appropriate shoes and socks
39	Help-seeking (when, where, how)
40	Stress management related to foot problems
Intervention tailoring	
41	Patients and family members who participate in the FIne-CHW educational sessions should be given healthy snacks if there is a sufficient budget
42	FIne-CHW sessions should be delivered in the local language

The statements were written in two languages: English and Indonesian. To minimise loss of meaning, the questionnaire was not employed using a back-and-forth translation since it is difficult to fully develop accurate and meaningful transcripts because the exact equivalence or meaning may not exist [12]. A bilingual registered nurse and the first author worked together to translate the Delphi questionnaire into Indonesian.

The statements were then transferred to the Jisc Online Surveys (formerly the Bristol Online Survey, Jisc, Bristol, UK). To ensure content and face validity, the questionnaire was piloted with three nurses in Indonesia who were not included in the panel before the survey was launched to all panellists. They were asked about the design, layout, clarity of information, and content. In general, they agreed that the survey was well presented, except for the logical flow of the survey on grouping statements on the same topic. The final revision was then carried out in consultation with the other authors of this paper.

The study was planned to have three rounds of Delphi consensus. During each round, participants were given two weeks to respond, with reminder emails sent at 7, 10, and 14 days. Panel members were asked to score the 42 statement core components of FIne-CHWs using a five-point Likert scale ranging from 1 to 5 (1 = strongly disagree, 2 = disagree, 3 = neither agree nor disagree, 4 = agree, 5 = strongly agree).

To increase the validity and credibility of the Delphi findings, Diamond et al. recommended that a priori criteria for success should be described. Consensus is typically determined by the percentage of agreement among participants, with the secondary factor being the proportion of participants who agreed within a particular rating range. The setting of the percentage level varies, but a 75% consensus rule-of-thumb is recommended to demonstrate the robustness of agreement [9]. Determining consensus was as follows: a statement was included in FIne-CHWs if it reached a consensus level of ≥75% agreement after three rounds, indicated by a score of four or five (strongly agree or agree); consensus agreement was determined by calculating the percentage of panel members combining the score of strongly agree and agree; statements were removed from FIne-CHWs if ≥75% of panel members scored them disagree or strongly disagree and for statements that did not reach consensus, the team reviewed and modified wording and then presented the revised statements to participants in the second round for assessment [9].

**Figure 1:**
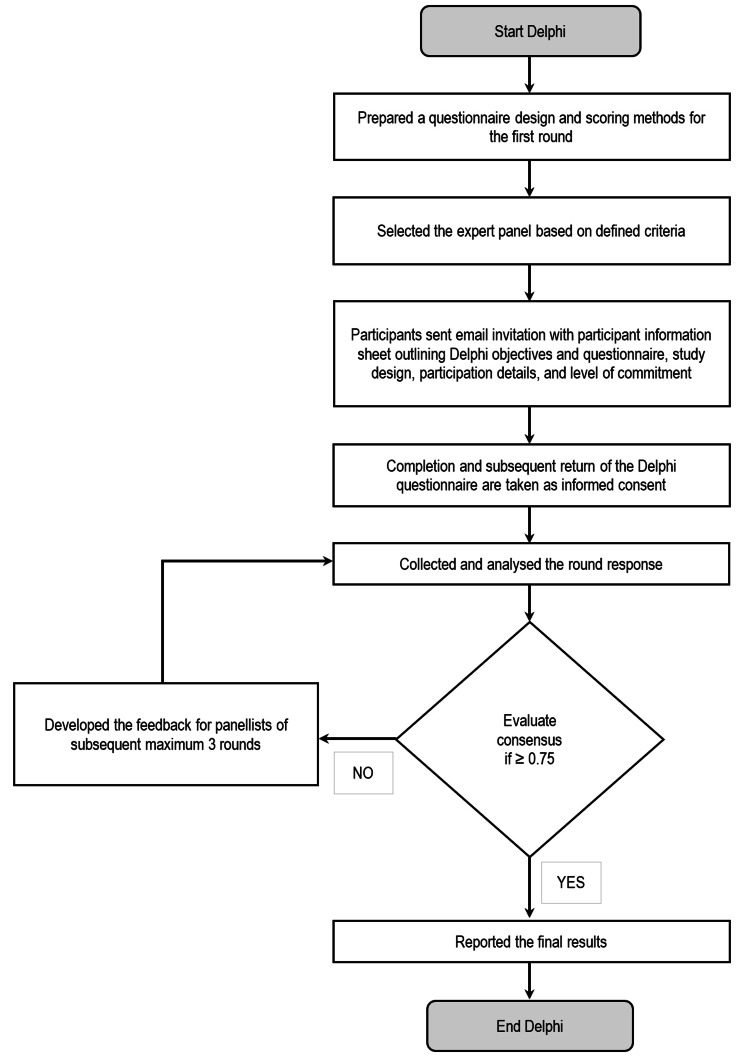
Modified Delphi study

In the subsequent round, participants got the chance to rethink and reconsider their decisions for each of the previous questionnaires or surveys and return their responses. This cycle of rounds continued until a point of diminishing return had been achieved, with a maximum of three rounds or no new information being forthcoming (Figure 1). After each round, statements that did not reach a consensus were reviewed by the research team. Revisions were made based on panellists' feedback, and these revised statements were recirculated in the subsequent round.

Selecting an expert panel based on defined criteria

Panel members were selected based on a minimum of five years of experience in diabetes management and a demonstrated history of publications or leadership in community health initiatives. A total of 28 panellists were chosen to ensure diverse representation from various regions and professional backgrounds. There are no specific guidelines on the number of individuals needed to constitute a representative sample. The minimum number of participants to ensure good group performance depends on the study design [13], and the quality of the panellists represents the quality of the feedback from those who know a particular area of ​​knowledge [14].

This study received ethics approval from the University of Nottingham Faculty of Medicine and Health Sciences Research Ethics Committee (no: FMHS 238-0421) and the Research Ethics Committee of Universitas Padjadjaran Bandung (no: 652/UNG.KEP/EC/2021).

Inclusion and Exclusion Criteria

Panel members were recruited through existing clinical (podiatrist, endocrinologist, medical rehabilitation specialist, and nurse wound specialist), academic, and professional bodies across Indonesia, including the Association of Community Nurses Indonesia (IPKKI), Indonesian Wound Ostomy Continence (InWOCNA), The Endocrinologist Society (PERKENI), Diabetes Educator Society (PEDI), Pencegahan dan Pengendalian Penyakit Tidak Menular Kementerian Kesehatan Republik Indonesia (P2PTM) Kementerian Kesehatan (Division of Non-Communicable Disease, MoH), Indonesia Diabetes Association (IDA/PERSADIA), and Rehabilitation Physical Medicine Association (PERDOSRI). We were not able to include CHWs on the panel of experts due to the lack of available CHWs' representatives at the national level. An invitation letter was sent to potential participants.

Recruitment Process

The first author emailed the professional organisations to identify panel members and asked them to recommend five experts. Two professional organisations (PERKENI and PERDOSRI) subsequently agreed to participate and provided contact information for five experts.

The gatekeeper from the MoH’s Division of Non-Communicable Disease (P2PTM) facilitated the first author contact with key individuals in the NCD programme, including the director of P2PTM (Division of Non-Communicable Disease, MoH), the head of the National Diabetes Programme, the former director of P2PTM, and the founder of the ISP-NCD in Indonesia, and researchers from the National Research and Innovation Agency (BRIN). These researchers, who had previously evaluated the ISP-NCD and diabetes in Indonesia, agreed to participate as panel experts. As not all organisational bodies responded, private emails were sent to experts. Some qualified experts from professional associations agreed to join the expert panel.

After contacting all participants through email or telephone, the first author enquired about their interest in participating. Those who expressed interest received a participant information sheet via email. Only those willing to complete all Delphi rounds were asked to participate. Completion and subsequent return of the Delphi survey were taken as informed consent (Figure 1).

## Results

Delphi results: first round

The initial round of the Delphi survey commenced on 18th May and concluded on 7th June 2022. Responses were received from 22 out of 28 panellists, which translates to a response rate of 78%. Four panellists did not reply to emails, and two panellists failed to respond even after the final reminder email, and subsequent private texts were sent. Characteristics of panellists are displayed in Table 2.

**Table 2:** Demographic data of panellists SD: standard deviation

Variable	N (%)
Profession	
Physician	11 (50%)
Nurse	9 (41%)
Pharmacist	1 (5%)
Epidemiologist	1 (5%)
Current job responsibilities	
Lecturer and published author in diabetes and community	7 (32%)
Lecturer and clinicians	6 (27%)
Health policy maker and analysis	5 (23%)
Clinicians	4 (18%)
Highest qualification	
Professor	1 (5%)
Doctorate	14 (64%)
Master	6 (27%)
Undergraduate	1 (5%)
Time qualified to work as a health professional/job (year)	
Mean ± SD	22.7 ± 6.1
Median	22
Range	16-40

Half of the participants (50%) were physicians working in academia as clinical lecturers, clinicians, or policymakers in the MoH, followed by 41% nurses who were lecturers in a community nursing department or nurse practitioners specialising in wound care. One pharmacist (5%) worked with research projects on diabetes, and one epidemiologist (5%) was a researcher affiliated with the MoH and was experienced in the evaluation of ISPs and CHW programmes (CHWPs) (Table 2).

All participants had considerable experience in managing diabetes in the community, with a mean of 22.7 years (± 6.1). Most of them graduated with a doctorate degree, and one member of the panel is the founder of the ISP-NCD, having 40 years of experience in policymaking in MoH and research institutes.

A total of 35 out of 42 statements reached a consensus agreement level of ≥75% in round one. One statement (item 1), which specified that only patients without an active DFU are eligible for the FIne-CHW sessions, reached a consensus of disagreement among the panel of experts and was consequently removed from consideration in the next round. The six statements that did not reach a consensus level of ≥75% agreement in round one were reviewed and refined by considering related open-text comments. These rephrased statements were re-presented to the panel in round 2.

Delphi results: second round

The second round of the Delphi survey took place over two weeks, from 17th June to 4th July 2022. To ensure that all panellists received the questionnaire, it was sent through two channels simultaneously: the Jisc Online Survey platform and email. This approach aimed to anticipate any issues where panellists may not have received the questionnaire through one of the channels. Of the 22 participants, 18 completed the survey, resulting in a response rate of 81%. All six statements reached the consensus level. These items were subsequently added to the core components of intervention FIne-CHWs.

Outcome of the Delphi Consensus on FIne-CHWs

Following two rounds of the Delphi exercise, consensus was reached on 41 out of 42 statements regarding the core components of the FIne-CHW programme. However, one statement (item 1), which specified that only patients without an active DFU are eligible for the FIne-CHW sessions, resulted in a consensus of disagreement and was, therefore, removed from the core components of the footcare intervention. The intervention components were defined and reported according to the TIDieR framework and categorised into themes: procedures, provider (CHWs or HCPs), place, method of delivery, time and duration, content, and tailoring [11].

Analysis data of the free-text responses

The modified Delphi survey used closed questions followed by open-ended questions to encourage participants to make comments on the topic in question. Data was generated through free text responses from the first and second rounds to obtain information, opinions, experiences, or practices. The analysis process followed six phases [15]. We began by familiarising ourselves with the dataset, reading text-based data items repeatedly to gain a thorough understanding of the data. Next, we explored the diversity and patterns within the datasets, developing codes and applying code labels to specific parts of the data through hand-coding. Developing initial themes allowed us to interpret the data in a descriptive manner for a comprehensive analysis. We then further explored these ideas in different contexts, such as community culture and health policy, using an inductive approach in theme development and review. Additionally, we ensured that each theme was clearly defined and contributed to the overall narrative of the overarching of data, resulting in a rich and nuanced analysis.

The overarching theme from the experts' panel on the consensus for CHWs delivering the FCI is "CHWs are only volunteers," reflecting the lack of policy for connectivity and integration of CHWs in healthcare service delivery.

Most participants emphasised the position of CHWs as volunteers who serve the community by encouraging them to participate actively in health programmes [6]. In Indonesia’s health system, CHWs are known as kader and also community members who manage the operations of ISP (Posyandu-Posbindu PTM) to be a forum to mobilise community participation for the success of community health programmes [6].

Some panellists narrated the position of CHWs in healthcare service delivery as volunteers and partners in the empowerment of the community participants to support health programmes:

What is meant by health centre (primary health centre (PHC)) management? Because kader are not subordinates/employees of the Puskesmas (PHC) but are partners and under supervision of the Puskesmas; referrals are made to Puskesmas, not to Posbindu PTM/posyandu (because) kader, are not professional. Kader are liaisons; kader work is done voluntarily, and transportation replacement is not a must. Kader are known laymen with being trained, not professionals, and so their relationship is with clients, not patients. (Panellist 22, physician, MoH)

The accounts highlight several limitations to the position of CHWs: CHWs are not PHC employees; as volunteers, incentives for their contribution to the community are not a must, and referring patients to CHWs is unacceptable because CHWs are not HCPs. The position of CHWs in terms of the relationship with the health system was also explained in the WHO reports that CHWs should be supported by the health system but not necessarily a part of its organisation [16]. Further arguments were narrated by several panellists who determined the CHW relationship with patients or members of the public:

Health kader are extra pairs of HCPs who are primarily driving the community-based health (UKBM) programmes (footcare education), which should be given by healthcare professionals. (Panellist 15, nurse educator, community nursing)

One panellist held the belief that CHWs are not in a position to educate the community. CHWs act as intermediaries between communities and health services, and at least part of their work consists of health education or health promotion, encouraging behavioural change. This role as a change agent (as a health promoter) is often undervalued by both governments and communities [17]. Clinicians were prominently concerned about whether CHWs would fully benefit patients despite their wide acceptance of the need for more diabetes prevention [18].

In Indonesia, the decentralised governance system makes ISP-NCDs (Posbindu PTM) activities highly dependent on the capacity and commitment of local governments, which impacts differences in the programme settings of CHWs [19]. On the other hand, the position of CHWs is functionally related to the delegation of tasks from HCPs. The activities of CHWs are under the management of the PHC while funding to support CHWs financially comes from the municipal council. In Indonesia, the nearest PHC provides technical guidance and support. The real accountability of the CHWs is to the village committee that appointed and supports them in their work [5]. One of the panellists was concerned about the sustainability of the programme if the PHC was to finance the CHWP.

What is needed is not a policy but a POB (community empowerment programme). Posbindu PTM is basically a community empowerment activity; if the financing is charged to the health facilities (PHC), then sustainability is difficult to maintain. (Panellist 14, physician, lecturer community medicine)

Overall, the panellists’ responses were situated within the milieu of a lack of clear regulations on how CHWs ought to be involved in care service delivery. This is despite CHWPs being part of national health initiatives, including components of CHW training integrated into MoH strategy. Clearly all CHWPs need further embeddedness, connectivity, and integration into the larger system of healthcare service delivery. Often the context and systems of which CHWs are a part, in particular, peripheral PHC services, are relatively neglected, providing a decontextualised picture of the role and function of CHWs [16]. No clear alignment of CHW to healthcare systems has the potential to impact the optimisation of FIne-CHWs, including clarity of CHW role, remuneration, and effect of implementation of programmes in the community. Across the three themes, this analysis extends the discussion of how this could affect FIne-CHW's implementation. Three themes are discussed in detail: (I) CHWs deliver footcare education for all diabetic patients (role CHW clarity), (II) remuneration and incentives, and (III) flexibility of community FCIs (community-based intervention).

Theme I: CHWs Deliver Footcare Education for All Diabetic Patients (CHW Role Clarity)

The role and position of CHWs are known as a bridge between HCPs and community members, helping communities identify and address their health needs independently [6]. Regarding footcare education, most of the panellists agreed that CHWs should be able to provide footcare education for diabetic patients with all stages of foot ulcers.

All patients with DM are at risk for the incidence of diabetic foot so all need to be educated. (Panellist 6, physician rehabilitation, physical medicine)

Several panellists raised concerns about CHWs' authority in managing NCDs in Indonesia, especially diabetic foot problems, due to the lack of regulations in the area.

The limits of kader’s authority must be determined so that if there are difficult topics, they just refer to the health workers. (Panellist 18, nurse educator, wound care specialist nurse)

The authority and responsibilities of CHWs were a concern for some panellists. Further consideration is needed to determine how FIne-CHWs fit with existing workloads.

Theme II: Remuneration and Incentives

Some concerns regarding remuneration or incentives were raised whereby CHWs were asked to conduct specific interventions outside of current duties; in such contexts, financial support to cover transportation costs was considered a minimum level of remuneration. One of the panellists narrated that the barrier faced by CHWs in serving the community is financial; she thought remunerating travel costs could be a reward for their hard work.

In addition, one of the panellists said that usually, a CHW is someone who needs financial support because they come from disadvantaged groups and cannot afford to incur additional costs to serve the community.

Those willing to become kader are usually/often someone who does not have a formal job and may belong to a low-income family group. If kader is willing to bear the cost of their own trip or the destination is within walking distance, it’s okay not to reimburse. (Panellist 3, physician rehabilitation, physical medicine)

Some panellists stated that CHWs should be reimbursed for transportation costs if they perform footcare education to motivate them. Satisfaction (or dissatisfaction) with incentives was closely linked to CHWs' motivation and performance [20]. One panellist argued that covering transportation costs would decrease CHWs' motivation and that non-monetary support such as providing lunch or having new uniforms provides a better appreciation of their effort.

The existence of kader is part of the active and voluntary participation of the community in health development. If they are required to provide transportation money, this will disrupt the motivation of the community to play an active role, because when there is money, kader will be active, but if there is no money, kader will not be active. Giving money can be replaced by eating lunch or giving uniforms or other incentives, according to the ability of the region. (Panellist 5, nurse educator, community nursing)

In summary, as noted by multiple panellists, the optimisation of CHWPs requires some kind of financial support, ranging from direct remuneration for transport costs they incur or in the form of gifts or food. Nonetheless, a crucial issue remains as to who bears responsibility for funding expenses related to CHWPs since there is no regulation in place regarding funding transportation costs. Policymakers should develop a policy (and funding) on this matter.

Theme III: Flexibility of Community FCIs (Community-Based Intervention)

Panellists reported that footcare education delivered by CHWs should be community-based and appropriate to community needs and resources. Health programmes involving CHWs are community empowerment involving community participants [20]. The intervention must be flexible to the context in which the intervention is implemented [21].

The problem of where interventions can be carried out was a concern among some panellists. One of the panellists narrated that multiple barriers were faced by patients to reach ISP-NCDs (Posbindu PTM).

It doesn’t have to be, because the problem that will also be faced is the community’s challenge to reach Posbindu PTM (ISP-NCD), not just motivation. Limited time, long distances, and an unsupportive economy are only some of the obstacles to the education programme, so implementation should not be rigid, and this is one of the reasons why it is important to educate kader in the same neighbourhood. (Panellist 6, physician rehabilitation, physical medicine)

Aside from the place of intervention, panellists also considered the flexibility of the education level of CHWs with regard to their potential role as intervention providers. Several panellists would accommodate CHWs below high school to deliver footcare education, as they thought that communication skills were more important than the level of formal education.

The main thing is the ability to communicate and communicate with the community. The level of education is often correlated with this ability. At least high school, because in some areas, it may be difficult to find kader who graduate higher than high school. (Panellist 13, pharmacist, lecturer)

The presence of HCPs as supervisors of CHWs when providing FCIs was not thought necessary by some panellists based on the assumption that CHWs are qualified volunteers who are sufficiently educated to deliver the education.

(The CHW/kader is) the extended hand of health workers (HCPs) who are more educated, not only at Posyandu, but also in daily activities. So, there is no need for strict supervision. (Panellist 18, nurse educator, wound care, specialist nurse)

## Discussion

Consensus was reached on 41 out of 42 statements regarding the core components of the FIne-CHWs programme. However, there was consensus disagreement on the eligibility criteria for patients to receive FIne-CHWs educational sessions, specifically concerning diabetic patients without DFUs. Most participants argued that all patients with all conditions should receive information regarding DFUs. In line with what the limitation of task sharing should be given to CHWs, this leads to further questions. What kind of actor is a CHW? The definition of CHWs as community volunteers was narrated by the panellists to respond to the statements that CHWs should be under the management of PHCs. This statement was made with the consideration that CHWs in Indonesia are involved in many ISPs with a wide range of roles, from the management of mothers and children to mental health posts [5]. However, panellists also noted that HCPs are not line managers of CHWs. This situation may unintentionally impact the introduction of new interventions, such as FIne-CHWs, which require increased involvement of HCPs in CHW management and coordination.

The statement that CHWs should be under the management of PHCs for delivering FIne-CHWs reached a consensus agreement. However, some panellists from the MoH argued that volunteers are not part of the health system organisation and have limitations regarding their role in serving the community; referring patients to FIne-CHWs was not feasible due to their non-health professional status and low-level positions within the healthcare system. Lehmann and Sanders stated that HCPs often perceive CHWs to be lowly aides, to be placed as assistants in health facilities, often misunderstanding their role in promoting and empowering health in society. In contrast, in many countries, CHWs have been set up to become agents of change in society; they function as extensions of formal healthcare, as auxiliaries rather than independent agents. This dilemma leads to the question of who owns and manages CHWPs and to whom CHWs are accountable [22].

In Indonesia, a high cultural value is placed on doing something for one’s neighbour without financial compensation except for a small reimbursement for expenses such as transportation costs. Volunteering as a CHW is greatly appreciated in the community. The roles of the MoH at the national level and its provincial agencies are largely responsible for setting norms and providing guidance to the lower-level administrators (PHCs) [5]. PHCs have no authority over financial and hiring/firing decisions related to CHWs, which are the responsibility of the village committees under the MoHA. The management of CHWs by two authorities (MoH and MoHA) impacts the CHWs’ position and unintentionally hinders optimising FIne-CHWs. Moreover, the absence of a direct hierarchy from PHCs influences the roles and functions of CHWs, which impacts FIne-CHWs’s implementation. This issued lack of policies was reported in CHWPs in Indonesia [5] and can lead to inadequate support for CHWs and CHWs not being recognised by health authorities, limiting how they operate in the community [23]. Moreover, it impacts the integration of CHWs into the broader healthcare system and the range of tasks that they perform, including referral and collaborative relationships with other HCPs in primary care teams [24]. Therefore, clear referral pathways are needed to ensure patients receive appropriate education and treatment where needed.

The statement that reached consensus disagreement pertained to the criteria for patients receiving footcare education sessions: "Only patients without active DFUs were eligible for FIne-CHW sessions." Most panellists thought that all diabetic patients with or without DFUs should be eligible to be taught by CHWs. All patients diagnosed with diabetes should be given clear information and explanation about DFUs, and early detection and treatment is one strategy to reduce the burden of diabetes [25,26]. Moreover, several studies have shown that CHW interventions in footcare education hold promise for improving foot self-care in diabetic patients [4]. However, the risk to patient safety of referring all diabetic patients without prerequisites to be managed by CHWs needs further consideration. CHWs are community volunteers with limited training and authority to perform specific tasks [24]. The delegation of roles from HCPs to CHWs can have a detrimental effect because the quality of care provided by CHWs can become less than optimal if CHWs are given complex tasks. Task sharing is an essential strategy for addressing shortages based on need, but quality and safety issues may arise, as well as professional and institutional resistance [20]. Protocols and guidelines can improve CHW performance because they help facilitate and coordinate CHW-related programmes such as role delegation. CHWs are unregistered healthcare workers with shorter training than HCPs; therefore, clear job descriptions should be defined in standard protocols to ensure the highest levels of CHW productivity [20].

Furthermore, one of the essential elements of a successful CHWP is CHW differentiation and role clarity to maintain a clear scope of work and accountability [27]. The lack of a well-defined and comprehensive definition for the position of CHWs within the healthcare system has resulted in a vague understanding of their role and responsibilities. This ambiguity poses a challenge when implementing new interventions such as FIne-CHWs and hampers the optimisation of CHWPs.

WHO released guidelines that follow a health systems approach and specifically identify the policy and system advocates needed to optimise the design and performance of CHW initiatives. The primary target audience for this guide is policymakers, planners, and managers responsible for health workforce policy and planning at national and local levels. These guidelines suggested using the following criteria for selecting CHWs for pre-service training, including the minimum level of education that must meet the task under consideration. The panellists concluded that the CHWs who provide footcare education should graduate from high school to be able to convey information to patients accurately. This requirement meets the manual for CHWs to run ISP-NCDs [6].

The Delphi statements also include personal attributes, capacities, values, and the professional experiences of the candidates (e.g., cognitive abilities, integrity, motivation, interpersonal skills, demonstrated commitment to community service, and a public service ethos) [24]. Additionally, they outline prerequisites for CHWs, such as the requirement that CHWs must not bring their personal political affiliations into their community service. This was considered a necessary precautionary measure to ensure acceptance by the wider community. CHWs need good interpersonal communication skills, good community engagement skills, and the opportunity to participate in community-based organisations [28].

Furthermore, CHWs must undergo footcare training, followed by an assessment of their knowledge, skills, and attitudes before they provide training to people; this requirement meets the recommendations of WHO on formal competency-based certification for CHWs who have successfully completed pre-service training and with priority emphasis on supervised practical experience in training CHWs [24].

The panellists also raised the issue of budget constraints to support CHWPs. There was little disagreement among the panellists about whether CHWs should be paid for their contributions to deliver FIne-CHWs. However, there are no regulations or budget resources available from PHCs and only a little funding from the village committee in exchange for CHWs’ role in managing community posts. This situation was identified when the government recognised CHWs, but the health system was still not equipped to monitor, support, and incentivise all the work of CHWs [24]. The provision of transportation costs for CHWs making patient home visits reached a consensus. This statement is in line with WHO's recommendations for remuneration of CHWs for their work, with a financial package commensurate with the demands of the work, complexity, number of hours, training, and the role they perform [24]. Motivation is sustained when CHWs feel they are valued members of the health system and have a clear role and set of responsibilities within it [29]. Providing multiple incentives over time has been shown to motivate CHWs in many successful programmes and can build a sense of satisfaction and ongoing satisfaction for CHWs, increasing their ability to work effectively [20].

Panellists raised concerns regarding the potential burden placed on eager and dependable volunteers who may become overwhelmed with additional tasks from PHCs (Puskesmas). This is due to the lack of alignment between the reimbursement and incentives provided by the village or local government, as well as the fact that CHWs are under the management of two authorities, namely the MoH and the MoHA. The effect of inadequate support can be that CHWs feel discredited by health authorities, which limits their ability to operate in the community [20].

Furthermore, the panellists argued that CHWPs should be run flexibly through local adaptation and take into consideration the available financial and human resources and physical facilities. It is widely recognised that there is a considerable gap between the ideals of community-driven and owned programmes and the reality of the programme with which CHWs can make a valuable contribution to community development [27]. Local geography (including proximity of households, distance to a clinic, and population density [24]) needs to be considered when choosing the site for the implementation of the FIne-CHW intervention. The panellists agreed that the sessions should be held in ISPs as they are places for community gatherings, close to an area neighbourhood so that the patients and CHWs do not require transportation costs to attend the education session.

CHWPs can achieve better integration into national initiatives when policymakers and community actors participate in CHWPs and view the programme positively [30]. A stronger CHW position in the health system is needed by defining clearly role and remuneration, as well as essential attributes, to support the CHWs' position in health service care delivery and the community. These contextual factors could challenge the feasibility of implementing the FIne-CHWs as a new structured educational intervention that has specific requirements to enhance CHWs' capacity to undertake this role.

The lack of policies on cohesiveness, connectivity, and integration into the wider healthcare delivery system can have impacts on the success of CHWPs [27], and it requires a functional system that allows CHWs to play a constructive role [22].

Strengths and limitations

The use of the modified Delphi technique allowed for systematic and iterative refinement of the intervention components, ensuring that the final consensus reflects a broad and informed perspective from a diverse panel of experts. Scholars believe that a modified two-step Delphi (close-ended) is an ideal approach to verifying the content and face validity compared to a traditional first round, which may create unambiguous, broad statements that could lead to bias from the outset [31]. Additionally, adhering to the TIDieR guidelines ensures that the intervention is described in sufficient detail, facilitating replication and adaptation in other settings [11].

Another strength of this study is the diversity of the expert panel, which included representatives from multiple disciplines and regions, thereby increasing the generalisability of the findings. Furthermore, the triangulation of data from scoping reviews, interviews, and expert consensus provides a robust foundation for the development of the intervention.

Although no CHWs were involved as panellists due to the lack of national-level representatives in Indonesia, their insights on footcare education programmes were gathered during prior interviews. These views were analysed and integrated into the core components of the intervention, which were later included in the Delphi consensus.

## Conclusions

The Delphi technique proved helpful in systematically obtaining consensus on 41 out of 42 core components of intervention FIne-CHWs, which means that the panellists agreed on specific core components of the intervention to be implemented in Indonesian contexts. The thematic analysis from the Delphi survey emerged one overarching theme: CHWs “are only volunteers.” This then was discussed in three themes, namely CHWs deliver footcare education for all people with diabetes, remuneration and incentives, and flexibility of community FCIs. However, to implement this intervention across the country, healthcare services authorities need to clarify the definition of CHWs’ function and role, including their position in health service delivery. Another issue with implementing FIne-CHWs in the real world is whether they can be standardised or whether they should maintain flexibility to be tailored according to specific local contexts.

The study's reliance on expert consensus, while valuable for identifying key components of the intervention, may not fully capture the complexities of implementing FIne-CHWs across varied healthcare settings. The perspectives of the panellists, although diverse, may not represent the views of all stakeholders, particularly those in under-represented regions or healthcare systems with limited resources. As such, further research involving broader stakeholder engagement and pilot testing is recommended before large-scale implementation.

## References

[1] International Diabetes Federation (IDF (2021). IDF Diabetes Atlas. https://diabetesatlas.org/.

[2] Yunir E, Tahapary DL, Tarigan TJ (2021). Non-vascular contributing factors of diabetic foot ulcer severity in national referral hospital of Indonesia. J Diabetes Metab Disord.

[3] Hariftyani AS, Novida H, Edward M (2021). Profile of diabetic foot ulcer patients at tertiary care hospital in Surabaya, Indonesia. J Berk Epidemiol.

[4] Okatiranti O, Nuryunarsih D, Windle R, Goldberg S (2024). Foot care intervention delivered by community health worker (CHWs): a scoping review. Glob Public Health.

[5] United States Agency for International Development (2020). Health for the people: national community health worker programs from Afghanistan to Zimbabwe. Health for the people: national community health worker programs from Afghanistan to Zimbabwe.

[6] Indonesian Ministry of Health (2012). Kementerian Kesehatan RI Direktorat Jenderal Pengendalian Penyakit dan Penyehatan Lingkungan Direktorat Pengendalian Penyakit Tidak Menular. Technical instructions for the integrated development post for non-communicable diseases (Posbindu PTM) (Book in Indonesian).

[7] Indonesian Ministry of Health (2019). Technical instructions for Posbindu integrated development post for kader.

[8] Okatiranti O (2024). Development of a footcare education intervention delivered by community health workers to Type 2 diabetes patients. Development of a footcare education intervention delivered by community health workers to Type 2 diabetes patients.

[9] Diamond IR, Grant RC, Feldman BM, Pencharz PB, Ling SC, Moore AM, Wales PW (2014). Defining consensus: a systematic review recommends methodologic criteria for reporting of Delphi studies. J Clin Epidemiol.

[10] Farmer T, Robinson K, Elliott SJ, Eyles J (2006). Developing and implementing a triangulation protocol for qualitative health research. Qual Health Res.

[11] Hoffmann TC, Glasziou PP, Boutron I (2014). Better reporting of interventions: template for intervention description and replication (TIDieR) checklist and guide. BMJ.

[12] Choi J, Kushner KE, Mill J, Lai DWL (2012). Understanding the language, the culture, and the experience: translation in cross-cultural research. Int J Qual Methods.

[13] Yousuf MI (2014). Using experts ’ opinions through Delphi technique. Pract Assesment Res Eval.

[14] Keeney S, Hasson F, McKenna HP (2010). The Delphi technique in nursing and health research. Wiley-Blackwell (ed.

[15] Braun V, Clarke V (2022). Thematic analysis: a practical guide. SAGE Publications Ltd (E-mail: info@sagepub.co.uk.

[16] Lehmann U, Sanders D (2007). Community health workers: What do we know about them? The state of the evidence on programmes, activities, costs and impact on health outcomes of using community health workers. WHO, Evidence and Information for Policy, Department of Human Resources for Health Geneva, January 2007: Community health workers: What do we know about them? The state of the evidence on programmes, activities, costs and impact on health outcomes of using community health workers. Uta Lehmann (ed).

[17] Perry HB, Hodgins S (2021). Health for the people: past, current, and future contributions of national community health worker programs to achieving global health goals. Glob Heal Sci Pract.

[18] Gore R, Brown A, Wong G, Sherman S, Schwartz M, Islam N (2020). Integrating community health workers into safety-net primary care for diabetes prevention: qualitative analysis of clinicians’ perspectives. J Gen Intern Med.

[19] (2021). Ministry of Home Affairs Regulation No 54 Year 2007 About Guidelines to Establish Operational Working Groups of Integrated Health Service Posts. https://dpmn.pasamanbaratkab.go.id/download/kategori/daftar-regulasi-kementerian-dalam-negeri.

[20] World Health Organization (2021). World Health Organization: What do we know about community health workers? A systematic review of existing reviews- Human Resources for Health Observer Series No 19. What do we know about community health workers? A systematic review of existing reviews.

[21] Skivington K, Matthews L, Simpson SA (2021). A new framework for developing and evaluating complex interventions: update of Medical Research Council guidance. BMJ.

[22] Hodgins S, Kok M, Musoke D, Lewin S, Crigler L, LeBan K, Perry HB (2021). Community health workers at the dawn of a new era: 1. Introduction: tensions confronting large-scale CHW programmes. Health Res Policy Syst.

[23] Kok MC, Kane SS, Tulloch O (2015). How does context influence performance of community health workers in low- and middle-income countries? Evidence from the literature. Health Res Policy Syst.

[24] World Health Organization (2018). World Health Organization (WHO): WHO Guideline on Health Policy And System Support to Optimize Community Health Worker Selected highlight. WHO guideline on health policy and system support to optimize community health worker programmes.

[25] National Institute for Health and Care Excellence (NICE) (2023). National Institute for Health Care Excellence (NICE): Diabetic foot problems: prevention and management. Public Health England, United Kingdom (ed). Diabetic foot problems: prevention and management.

[26] International Diabetes Federation (2021). International Diabetes Federation (IDF): clinical practice recommendations on the diabetic foot -International Diabetes Federation, Brussels Belgium. Clinical practice recommendations on the diabetic foot.

[27] Lehmann U, Twum-Danso NA, Nyoni J (2019). Towards universal health coverage: what are the system requirements for effective large-scale community health worker programmes?. BMJ Glob Health.

[28] LeBan K, Kok M, Perry HB (2021). Community health workers at the dawn of a new era: 9. CHWs' relationships with the health system and communities. Health Res Policy Syst.

[29] Colvin CJ, Hodgins S, Perry HB (2021). Community health workers at the dawn of a new era: 8. Incentives and remuneration. Health Res Policy Syst.

[30] Zulu JM, Kinsman J, Michelo C, Hurtig AK (2014). Integrating national community-based health worker programmes into health systems: a systematic review identifying lessons learned from low-and middle-income countries. BMC Public Health.

[31] Hasson F, Keeney S (2011). Enhancing rigour in the Delphi technique research. Technol Forecast Soc Change.

